# 905. Epidemiology of Enterovirus D68 in the US: New Vaccine Surveillance Network, 2017–2022

**DOI:** 10.1093/ofid/ofad500.950

**Published:** 2023-11-27

**Authors:** Benjamin R Clopper, Heidi L Moline, Claire Midgley, Adriana S Lopez, Terry Fei Fan Ng, Ariana Perez, Mary A Staat, Elizabeth P Schlaudecker, Leila C Sahni, Julie A Boom, Jennifer E Schuster, Rangaraj Selvarangan, Natasha B Halasa, Laura S Stewart, Robert Hickey, Marian G Michaels, Geoffrey A Weinberg, Peter G Szilagyi, Eileen J Klein, Janet A Englund

**Affiliations:** US Centers for Disease Control & Prevention, Buffalo, New York; Centers for Disease Control and Prevention, Atlanta, Georgia; Centers for Disease Control and Prevention, Atlanta, Georgia; Centers for Disease Control, Atlanta, Georgia; CDC, Atlanta, Georgia; CDC, Atlanta, Georgia; Cincinnati Children’s Hospital Medical Center, Cincinnati, Ohio; Cincinnati Children's Hospital Medical Center, Cincinnati, Ohio; Baylor College of Medicine and Texas Children’s Hospital, Houston, Texas; Texas Children’s Hospital, Houston, Texas; Children’s Mercy Kansas City, Kansas City, Missouri; Children’s Mercy Kansas City, Kansas City, Missouri; Vanderbilt University Medical Center, Nashville, Tennessee; Vanderbilt University Medical Center, Nashville, Tennessee; Childrens Hospital of Pittsburgh, Pittsburgh, Pennsylvania; UPMC Children's Hospital of Pittsburgh, Pittsburgh, Pennsylvania; University of Rochester School of Medicine & Dentistry, Rochester, NY; UCLA School of Medicine, Agoura Hills, California; University of Washington School of Medicine, Seattle, Washington; Seattle Children’s Hospital, Seattle, Washington

## Abstract

**Background:**

Provisional US data indicated that enterovirus D68 (EV-D68) circulated during summer 2022. However, in contrast to 2018 (a previous high circulation year), EV-D68 circulation in 2022 was associated with unusual increases in asthma-specific healthcare visits and a lack of concomitant increases in acute flaccid myelitis (AFM). To explore these distinctions by year, we characterized respiratory EV-D68 circulation in 2022 and compared patient characteristics in 2022 to 2018.

**Methods:**

We enrolled children aged < 18 years with acute respiratory illness (ARI) seeking care in an emergency department (ED) or as an inpatient (IP) across 7 US medical centers in the New Vaccine Surveillance Network (NVSN). Data sources included parent interview, medical chart review, and collection of a respiratory swab for molecular virus testing. Swabs were tested for EV-D68 from Jul–Nov 2017–2020, and year-round from Jul 2021. A convenience sample of EV-D68-positive swabs was sequenced. We assessed monthly EV-D68 percent positivity among children with ARI. We examined demographics, underlying conditions, and severity markers among children with EV-D68 in 2018 and 2022, by care setting (ED vs IP) and used 0.05 as a threshold of statistical significance.

**Results:**

Between 2017–2022, there were 994 ED and IP EV-D68 detections at NVSN sites, with distinct peaks during the Jul-Nov testing periods in 2018 and 2022 (**Figure**). All viruses sequenced in 2018 and 2022 were lineage B3. Compared to 2018, IPs with EV-D68 in 2022 less frequently reported any underlying medical condition or a history of asthma, yet more frequently required supplemental oxygen or intubation (**Table**). Supplemental oxygen use was also more common among ED patients in 2022 than 2018 (13% vs 6%; p=0.028). Asthma exacerbation was a common primary discharge diagnosis among IPs but was less common in 2022 than 2018 (43% vs 53%; p=0.030).

**Figure:**
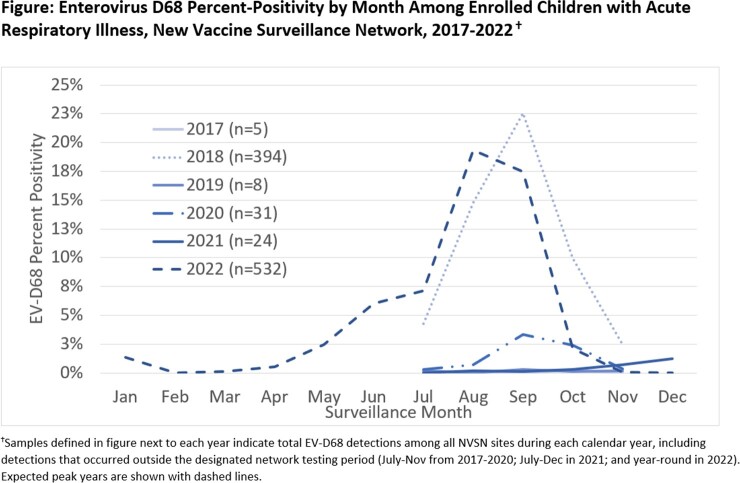

**Figure:**
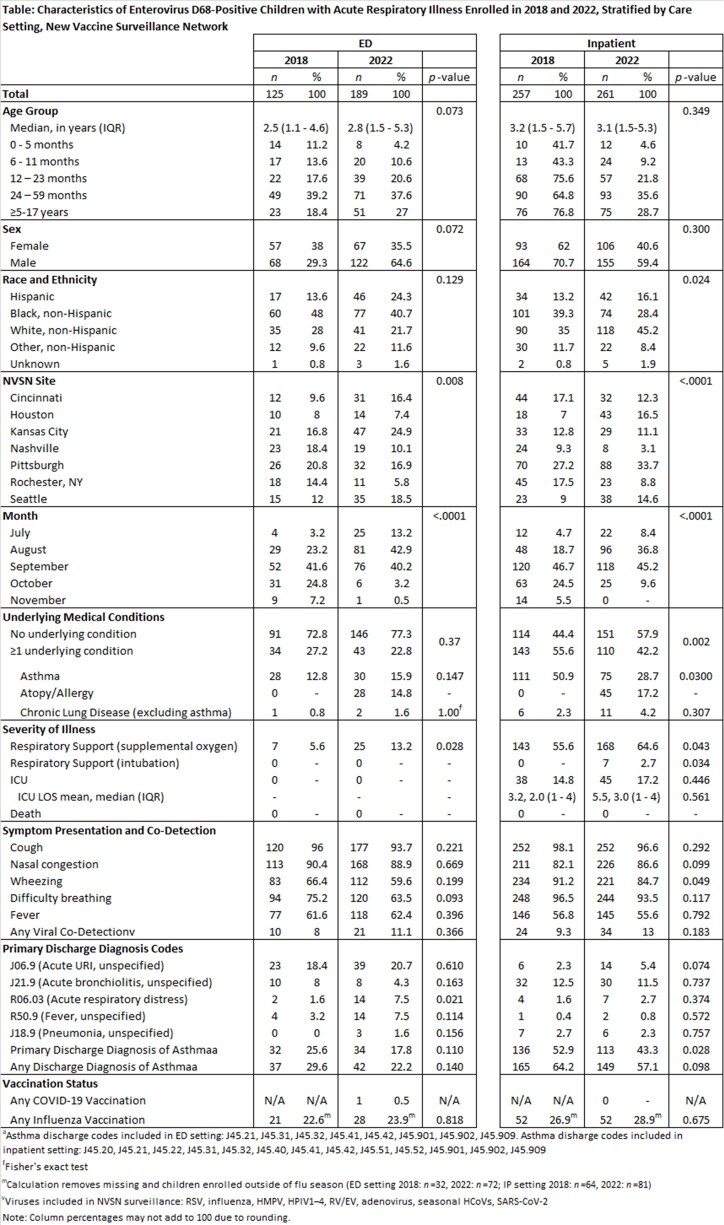

**Conclusion:**

EV-D68 circulation was high in 2018, appeared to be disrupted in 2020, and returned with early and high circulation in 2022. Compared to 2018, EV-D68 may have caused more severe respiratory disease in 2022, including in otherwise healthy children. The lack of AFM observed in 2022 despite high EV-D68 circulation needs further investigation.

**Disclosures:**

**Mary A. Staat, MD, MPH**, CDC: Grant/Research Support|Cepheid: Grant/Research Support|Merck: Grant/Research Support|NIH: Grant/Research Support|Pfizer: Grant/Research Support|Up-To-Date: Honoraria **Elizabeth P. Schlaudecker, MD, MPH**, Pfizer: Grant/Research Support|Sanofi Pasteur: Advisor/Consultant **Rangaraj Selvarangan, BVSc, PhD, D(ABMM), FIDSA, FAAM**, Abbott: Honoraria|Altona Diagnostics: Grant/Research Support|Baebies Inc: Advisor/Consultant|BioMerieux: Advisor/Consultant|BioMerieux: Grant/Research Support|Bio-Rad: Grant/Research Support|Cepheid: Grant/Research Support|GSK: Advisor/Consultant|Hologic: Grant/Research Support|Lab Simply: Advisor/Consultant|Luminex: Grant/Research Support **Natasha B. Halasa, MD, MPH**, Merck: Grant/Research Support|Quidell: Grant/Research Support|Quidell: donation of kits|Sanofi: Grant/Research Support|Sanofi: vaccine support **Marian G. Michaels, MD, MPH**, Merck: Grant/Research Support|Viracor: Grant/Research Support **Geoffrey A. Weinberg, MD**, Merck & Co: Honoraria **Janet A. Englund, MD**, Ark Biopharma: Advisor/Consultant|AstraZeneca: Advisor/Consultant|AstraZeneca: Grant/Research Support|GlaxoSmithKline: Grant/Research Support|Meissa Vaccines: Advisor/Consultant|Merck: Grant/Research Support|Moderna: Advisor/Consultant|Moderna: Grant/Research Support|Pfizer: Advisor/Consultant|Pfizer: Grant/Research Support|Sanofi Pasteur: Advisor/Consultant

